# Caring as a Generative Principle: Reconfiguring the Metaparadigm and Operative Mechanisms in Nursing Theory

**DOI:** 10.1111/jan.70544

**Published:** 2026-02-15

**Authors:** Aarón Muñoz Devesa, Juan Ignacio Rico Becerra

**Affiliations:** ^1^ Faculty of Health and Social Sciences University of Murcia Lorca Spain

**Keywords:** caring, meta‐theory, nursing metaparadigm, nursing theory, philosophy of nursing

## Abstract

**Aim(s):**

To develop a comparative meta‐theory of nine caring theories by explicating their assumptions, operative mechanisms and consequences for nursing.

**Design:**

Qualitative meta‐theoretical document analysis.

**Methods:**

Canonical texts were analysed using an intra‐source strategy. Paginated statements were extracted and coded across assumptions, metaparadigm anchors (person, health, environment, nursing and care) and mechanisms linking caring intention to clinical action. Synthesis produced a typology and meta‐theoretical propositions.

**Results:**

Caring functioned as a generative principle that reorganised person, health, environment and nursing and care into distinct practice architectures. Six mechanism‐based subfamilies were identified: transpersonal caritas; phenomenological and embodied clinical wisdom; ethical and relational caring; cultural and contextual caring; systemic and organisational caring; and operationalisable caring. Ten propositions linked assumptions to mechanisms and expected effects.

**Conclusion:**

The caring school is best understood as an ordered set of non‐equivalent caring mechanisms rather than a single doctrine, supporting translation to practice design, education and congruent evaluation.

**Implications for the Profession and/or Patient Care:**

Mechanism‐based comparison can reduce conceptual ambiguity and improve alignment between caring interventions and intended outcomes.

**Impact:**

This study addresses the under‐specification of how caring theories work. It provides a comparative typology and propositions that make mechanisms explicit, informing nursing education, theory development and caring‐based practice in diverse settings.

**Reporting Method:**

No EQUATOR reporting checklist is available for meta‐theoretical discursive analyses; the manuscript follows Journal of Advanced Nursing guidance for discursive papers.

**Patient or Public Contribution:**

This study did not include patient or public involvement in its design, conduct, or reporting.

## What Does This Paper Contribute to the Wider Global Clinical Community?

- Reframes caring as an operator that reshapes core nursing concepts.
- Provides a mechanism‐based typology across nine influential caring theories.
- Offers comparative propositions to strengthen theory‐consistent practice and evaluation.

## 1 Introduction

In recent decades, nursing has explicitly reinforced its disciplinary identity around caring as a central phenomenon. However, the term's diffusion across education, clinical practice, research and management has had an ambivalent effect: the more caring is invoked as the discipline's core, the more it risks functioning as an all‐purpose signifier—capable of covering almost any professional action and therefore losing heuristic power to specify assumptions, mechanisms and consequences.

This is not a minor issue. Conceptual scholarship has shown that ‘caring’ can function as a moral ideal, a relational mode, a clinical process, a cultural framework or an organisational category. Such semantic plurality demands metatheoretical systematisation if the concept is to remain scientifically useful rather than degrading into a totalising term that cannot differentiate professional consequences (Morse et al. 1990; Finfgeld‐Connett 2008).

Within this landscape, the school of caring considered here (Watson; Boykin & Schoenhofer; Eriksson; Benner; Martinsen; Ray; Leininger; Swanson; and Kolcaba) is both fertile and conceptually demanding. It is fertile because it offers architectures that reposition care as a human, ethical and relational practice; it is demanding because it gathers non‐interchangeable ontologies, epistemologies and ethics under a single label.

Across these theories, some frameworks privilege transpersonal or caritative horizons; others foreground phenomenological, embodied and situated understandings; others problematise power, systems and organisational rationalities; others make cultural congruence constitutive; and others translate caring into evaluable outcomes such as comfort. This plurality is a disciplinary strength provided that constitutive differences are made explicit, thereby preventing reduction to a ‘lowest common denominator’ version of caring (Morse et al. 1990; Finfgeld‐Connett 2008).

From the standpoint of nursing theory, a controlled comparison cannot be reduced to narrative synthesis. The metatheoretical problem is how to identify convergences without erasing irreducible tensions. This question points to the level at which the discipline organises its thinking: the nursing metaparadigm (person, health, environment and nursing/care) and the relations that articulate it. Metaparadigmatic debates remain relevant because these definitions are not neutral; they shape what is taken to be real in care, what counts as legitimate knowledge and what is expected as an outcome of practice (Fawcett 1984; Bender 2018).

Conceptual precision becomes even more pressing under contemporary pressures of standardisation, productivity, documentation, technologisation and organisational rationality. These conditions can erode the moral integrity of practice and contribute to moral distress and burnout when nurses perceive a gap between what care ought to be and what systems allow them to do (Salas‐Bergüés et al. 2024). In parallel, increasingly digital care environments require a reconsideration of whether technology reduces care to instrumentality or can, under specific conditions, mediate caring practices (Nakano et al. 2021). To compare these frameworks under controlled conditions, we used Modelos y teorías en enfermería as the base text because it offers a delimited corpus presented with a broadly standardised chapter architecture, supporting systematic extraction of comparable definitions and assumptions and enabling a shared metaparadigmatic matrix for analysis (Alligood 2022).

### 1.1 Aim

This study aimed to develop a comparative metatheory of nine caring theories/models presented as the school of caring in Modelos y teorías en enfermería (Alligood 2022)—Watson; Boykin and Schoenhofer; Eriksson; Benner; Martinsen; Ray; Leininger; Swanson; and Kolcaba—by: (a) systematically comparing their ontological, epistemological and ethical assumptions; (b) analysing their conceptualisations of the nursing metaparadigm (person, health, environment and nursing/care); and (c) constructing an integrative metatheoretical model and a set of metatheoretical propositions that make explicit convergences, non‐reducible tensions and implications for nursing practice, education and research.

## 2 Methods

### 2.1 Design

We conducted a qualitative metatheoretical document analysis to analyse, compare and integrate caring theories/models as presented in Modelos y teorías en enfermería (Alligood 2022; 10th Spanish edition). The aim was to generate (i) an integrative metatheoretical model and (ii) a set of metatheoretical propositions that specify relations between metaparadigmatic concepts (person, health, environment and nursing/care) and underlying assumptions (ontological, epistemological and ethical). Metatheory is not limited to summarising theories; it interrogates conceptual language, internal structure and inferential logic to formulate higher‐order relational statements of disciplinary utility (King and Fawcett 1997).

The study used an intra‐source strategy: the nine theories/models of the school of caring were analysed as they are canonically presented within the same reference text. This epistemological decision ensured comparative consistency, traceability and control of editorial variance, so that observed differences are attributable primarily to theoretical distinctions rather than heterogeneity in presentation (Bowen 2009).

### 2.2 Base Text/Intra‐Source Corpus and Inclusion Criteria

The base text/intra‐source corpus consisted exclusively of nine nursing theories/models presented as part of the school of caring in the 10th edition of *Nursing Theorists and Their Work* (Alligood 2022). These theories were: Jean Watson, Anne Boykin and Savina O. Schoenhofer, Katie Eriksson, Patricia Benner, Kari Martinsen, Marilyn Anne Ray, Madeleine M. Leininger, Kristen M. Swanson and Katharine Kolcaba.

Inclusion was based on explicit editorial and conceptual criteria: (a) that care (caring) was presented as the central and defining phenomenon of the discipline within the corresponding chapter and (b) that the theory offered explicit or inferable formulations of the metaparadigm (person, health, environment and nursing/care), as well as the assumptions and mechanisms of caring. Other models in the manual that, while relevant to the discipline, do not conceptualise care as the primary structuring axis were not included.

The delimitation of the corpus to these nine theories responds to a criterion of comparative consistency, as they all share a homogeneous presentation architecture within the same base text, allowing for a controlled metatheoretical comparison without introducing biases derived from heterogeneous editorial sources.

### 2.3 Intra‐Source Verification Strategy

To reinforce interpretive coherence within the intra‐source design, an internal verification strategy based exclusively on Alligood (2022) was applied. This strategy does not seek to contrast historical fidelity regarding the original works, but rather to ensure comparative consistency and traceability of inferences within the base text. Minimum rules were applied: (a) every metatheoretical statement (assumption, definition, mechanism, or metaparadigmatic relationship) was anchored in textual evidence with pagination in Tables A1, A2, A3, A4, A5; (b) when a formulation was synthetic or potentially ambiguous, it was verified through intra‐source corroboration (e.g., contrast between definition, metaparadigmatic section and operational descriptors of the same chapter) to avoid extrapolations; (c) interpretation was restricted to what was explicitly or inferably codified by the base text, avoiding the importation of terminology or developments not present in Alligood (2022). Thus, the robustness of the analysis stems from the transparency of the textual trail and the control of editorial variance, consistent with the purpose of the study.

### 2.4 Metatheoretical Analytical Framework

The analysis framework was structured into three levels, consistent with metatheoretical comparison:

Level of Assumptions: ontology (what is the person/reality of care), epistemology (what counts as valid knowledge) and ethics (what grounds the obligation to care).

Level of Concepts: conceptual operationalisation of the metaparadigm (person, health, environment, nursing/care), following the disciplinary relevance of the metaparadigm as an organiser of theories and theoretical debate in nursing (Fawcett 1984).

Relational/Propositional Level: identification of relationships such as “under X conceptual/contextual conditions → caring adopts Y form → produces Z expected effects” to derive comparable propositions between theories.

### 2.5 Data Extraction and Organisation Procedure

An extraction matrix was designed in Excel (unit of analysis: textual fragment with theoretical meaning), recording for each theory: definitions, attributes, assumptions, processual components of caring, relationships between concepts and analytical notes. In this phase, a documentary analysis procedure was applied with iterative reading, selection of relevant units and systematic organisation of textual evidence (Bowen 2009).

### 2.6 Coding and Synthesis Strategy

Qualitative content analysis was used, combining deductive and abductive‐inductive components. Deductive coding started from an initial scheme based on the metaparadigm (person, health, environment, nursing/care) and metatheoretical dimensions (ontological/epistemological/ethical assumptions). This approach aligns with the logic of directed content analysis, which starts from a priori categories and allows for extensions derived from the data (Hsieh and Shannon 2005).

Complementarily, emergent codes were incorporated to capture constitutive differences (e.g., transpersonality, *caritas*, *phronesis*, relational vulnerability, institutional critique, culture, bureaucracy, comfort, etc.). The process followed the typical phases of content analysis (preparation, organisation and abstraction) described for qualitative research applied to texts (Elo and Kyngäs 2008).

### 2.7 Coding by Two Analysts and Consensus Strategy

Two researchers carried out the coding independently on an initial subset of the corpus (≈20%) for the purpose of calibrating the analytical framework, refining operational definitions of codes and agreeing on decision rules. Following this calibration phase, a codebook was consolidated and changes were documented in an audit trail. Subsequently, the full corpus was coded following the agreed codebook, and discrepancies were resolved through consensus in comparison sessions, jointly reviewing fragments and justifying coding decisions based on textual evidence and established rules. The final agreements and modifications were incorporated into the audit trail, prioritising a qualitative consensus strategy oriented towards interpretive coherence and process transparency (O'Connor and Joffe 2020).

### 2.8 Construction of the Metatheoretical Model and Formulation of Propositions

The metatheoretical synthesis was developed into two products:

Integrative Metatheoretical Model: articulation of convergences (conceptual invariants) and divergences (irreducible tensions) between theories, organised in a common explanatory scheme.

Metatheoretical Propositions: relational statements derived from systematic comparison, formulated to be debatable, transferable and useful for practice, teaching and research.

The construction of these products was supported by classic strategies of theoretical development/structuring in nursing, particularly the logic of moving from concepts to relationships and from relationships to comparable propositions (Walker and Avant 2019).

### 2.9 Criteria of Rigour (Trustworthiness)

Criteria of credibility, transferability, dependability and confirmability were applied to reinforce the rigour of the qualitative analysis (Lincoln and Guba 1985). Specifically: (a) credibility through independent coding and contrast between analysts, with resolution by consensus; (b) dependability through the recording of codebook changes and decision auditing; (c) confirmability through the traceability of statements to textual evidence with pagination and agreements; and (d) transferability through the explicit description of the corpus, criteria and study limits.

### 2.10 Ethical Considerations and Use of AI

No human participants or clinical data were involved; the study used exclusively bibliographic and documentary sources; therefore it did not require ethical evaluation for research involving human subjects. Compliance with citation standards and copyright was maintained. The translations of textual quotes in English are the responsibility of the author of this work.

Regarding the use of artificial intelligence, it was employed as support for drafting, editing and translation, with the authors maintaining full responsibility for the final content at all times. The use of AI will be declared transparently according to editorial ethics recommendations: AI tools must not be listed as authors, and the authors remain responsible for the manuscript (COPE 2023).

## 3 Results

Given the metatheoretical nature of the design, the results are presented as interpretive structures derived from a systematic documentary analysis (comparative patterns, typologies and relational propositions), maintaining traceability between textual fragments, analytical categories and the resulting metatheoretical syntheses.

The results are supported by Tables A1, A2, A3, A4, A5, which contain identifying fragments with printed pagination, allowing for the verification of the minimum evidence used in the text. Full verbatim quotes and analytical notes are preserved in extraction matrices. Consequently, this section reports findings as comparative patterns and provides sufficient minimum evidence through representative examples selected for their conceptual clarity and their ability to illustrate the described pattern (Alligood 2022, 74, 88, 109, 131, 148–150, 294–295, 313, 340–342, 528, 530, 556–557). The subfamily designations used below correspond to analytical labels derived from the coding and synthesis process.

### 3.1 Operational Definitions of Caring by Theory/Model

Table A1 shows that caring is a nuclear concept in the nine theories/models analysed, but its operational definition is not univocal. The primary comparative finding is a structural convergence—care as a moral‐relational reality irreducible to technique—alongside a stable semantic divergence organised into distinguishable families (Table A1).

In the analysed corpus, definitions are grouped into six taxonomically differentiated semantic families (Table A1). In the transpersonal/caritas family, care is enunciated from a moral‐spiritual horizon, formulated as holistic presence/relationship (Watson) and as care animated by *caritas* oriented towards suffering (Eriksson). In the phenomenological‐embodied/clinical wisdom family, caring is linked to the concrete encounter and to practical knowledge or *phronesis*: Benner expresses it as connection and concern that enables help, and Martinsen as a basal act oriented towards the situation of the other. This configuration positions practical competence not as an adjunct technique, but as a situated operation of ethical perception. In the ethical‐relational family, Boykin and Schoenhofer place care as an altruistic and active expression linked to intentionally knowing the value of the other within the relationship. In the cultural‐contextual family, Leininger positions care as a dominant and distinctive epistemological construct, underlining cultural congruence. In the systemic‐organisational family, Ray integrates care with normative and bureaucratic mediations.

Finally, in the operationalisable caring family (F), Kolcaba and Swanson employ pragmatic formulations oriented towards the translation of care into processes and outcomes; in Swanson, caring is defined as a nurturing relationship, facilitating its integration into management processes and outcome evaluation (Table A1).

In comparative terms, this definitional variation is not merely terminological: it anticipates stable differences in the purpose (*telos*), in the status of the environment and in the way each theory makes caring “operable” (processes, mediations, or outputs), which justifies its integrated analysis in the following sections.

### 3.2 Purpose or *Telos* of Caring

Table A2 shows that the purpose attributed to care robustly discriminates between theories/models: caring is not only defined differently but is also oriented towards different goods/ends, with direct implications for practice, education and research (Table A2).

In the analysed corpus, dominant teleological orientations are observed, acting as axes for the validation of practice (Table A2). In Watson, the *telos* is linked to processes of meaning, human healing and transformation of experience; in Eriksson, to the relief of suffering through communion and assistance animated by *caritas*. In Leininger, the purpose is articulated as culturally congruent well‐being/health, while in Swanson, it is associated with dignity, respect and empowerment. In Kolcaba, it is formulated in terms of comfort to facilitate health‐seeking behaviours chosen by the person. Benner and Martinsen enunciate the *telos* in terms of the possibility of help in a given situation, while Boykin and Schoenhofer express it as living and growing in the context of care; Ray formulates it as a choice oriented towards the good of the other under institutional mediations. This teleological diversity confirms that health is not a neutral end, but a normatively oriented category that legitimises different indicators of success according to the applied framework.

### 3.3 Metaparadigmatic Anchors: Person, Health, Environment and Nursing/Care

Table A3 highlights that the four metaparadigmatic concepts do not operate as parallel categories but as a system whose internal logic is reconfigured by caring. The most stable common pattern is that nursing/care is presented as a relational praxis with moral density, transcending mere technical intervention. The comparison, however, reveals systematic variations in the ontology of the person and in the status of health, alongside an especially marked variation in the environment, which emerges as the primary metaparadigmatic discriminant due to its drastic change in scale.

In this gradient, the environment shifts from a broad and universal‐spiritual horizon in Watson to being formulated as a significant situation and a lived space of encounter in the frameworks of Benner and Martinsen. For her part, in Leininger's theory, the environment becomes inseparable from the cultural context, while in Ray, it incorporates organisational and normative mediations, including bureaucratic and technological structures that condition practice. Finally, in Kolcaba's model, the environment operates as a dimension susceptible to intervention and manipulation oriented towards optimising comfort and obtaining observable effects.

This gradient of scale in the environment corresponds to differentiated forms of care—transpersonal presence, situated clinical prudence, cultural congruence, systemic mediation, or operationalisation—which become fully visible when comparing the four metaparadigmatic components for each theory.

### 3.4 Operational Mechanisms: From Intention to Clinical Action

Table A5 identifies the mechanisms through which caring is translated into clinical action, organising them into five functional families that act as the operative engine of each theoretical framework. These mechanisms vary from explicit processes (F1), which offer operational structures and intentional guides as in Watson, Swanson, Leininger and Kolcaba, to institutional mediations (F2) proposed by Ray, where care is negotiated within bureaucratic and technological systems. Likewise, situated epistemic‐moral operations (F3) are described in Benner and Martinsen, based on clinical judgement and ethical perception, alongside mechanisms centred on the confirmation of suffering (F4) in Eriksson, or the “nursing situation” as the minimum analytical unit (F5) in Boykin and Schoenhofer (Alligood 2022, 74, 86, 113, 132, 150, 292, 342, 535, 556).

This diversity of devices confirms that caring operates through differentiated logics of discernment and mediation, reinforcing the thesis of the “ordered plurality” of the school. To ensure the robustness of this classification, Table A5 presents, for each theory: (a) the dominant mechanism, (b) its synthetic formulation and (c) the nuclear supporting fragment extracted directly from the base text with its respective printed pagination.

#### 3.4.1 Explicit Processes: When Caring Is Formulated as an Operational Structure

Four theories express formalised mechanisms (sequences, modes of action, or taxonomic structures) that make caring describable and, to varying degrees, translatable into teaching and evaluation. In Watson, the mechanism is articulated through *caritas* processes; these are presented as essential care and guide care as an intentional encounter sustained by moral and relational consciousness (Alligood 2022, 74). In Swanson, the mechanism is expressed in relational processes that guide action, where “being with” is defined as emotional presence and “maintaining belief” as sustaining faith in the other's capacity (Alligood 2022, 556). In Leininger, the mechanism operates as the cultural translation of care through three modes of action—preservation/maintenance, accommodation/negotiation and restructuring/repatterning—which organise culturally congruent care (Alligood 2022, 342). In Kolcaba, the mechanism is formulated through a taxonomic structure of comfort that explicates states of comfort as an outcome of care (relief, ease and transcendence) (Alligood 2022, 535).

#### 3.4.2 Institutional Mediations: Caring as Moral Action Within the System

In Ray, the mechanism of care is defined by its articulation with technological and bureaucratic structures. The theory explicates dimensions of *bureaucratic caring*—ethical‐religious, humanistic, economic, political, legal, technological and educational—which function as mediators of care (Alligood 2022, 86). In this framework, the moral quality of caring depends on how human/spiritual values are sustained and negotiated within the system.

#### 3.4.3 Situated Epistemic‐Moral Operations: Clinical Judgement and Perception as Mechanism

Benner and Martinsen provide mechanisms that are not formulated as lists of steps, but as situated operations that integrate knowledge, morality and clinical response. In Benner, expert care is supported by clinical judgement and embodied practical capacity; clinical judgement is described as the mechanism that allows for the interpretation of the situation, discernment of priorities and appropriate response in concrete contexts (Alligood 2022, 113). In Martinsen, the mechanism is articulated through perception and attention: ethical perception—sensitive to the situation and the other—opens the possibility of care within the relationship (Alligood 2022, 132).

#### 3.4.4 Suffering and the Charitable Relationship: The Confirmation Mechanism

In Eriksson, the central mechanism is formulated around suffering and its responsible recognition. Care includes the “confirmation of the patient's suffering” (Alligood 2022, 150). In this framework, caring operates by confirming the reality of suffering as a constitutive act of charitable care, with ethical‐existential outputs linked to dignity and relief.

#### 3.4.5 Minimum Analytical Unit: The “Nursing Situation” as the Deployment of Caring

Boykin and Schoenhofer reconfigure the unit of care analysis: the “nursing situation” is defined as the expression of values, intentions and actions of two or more persons (Alligood 2022, 292). This result defines caring as a co‐produced relational event and offers a minimum analytical unit for describing care beyond unilateral action.

### 3.5 Internal Typology: Subfamilies and Dominant Mechanism of Caring

Integrating definitions (Table A1), *telos* (Table A2), metaparadigmatic anchors (Table A3) and operational mechanisms of care (Table A5), Table A4 organises the school of caring into an internal typology of six subfamilies. This result allows the school of caring to be described as a theoretical family with recognisable convergences and structural divergences, rather than a general terminological label (Table A4).

The typology distinguishes: (A) transpersonal/caritas (Watson; Eriksson), (B) phenomenological‐embodied/clinical wisdom (Benner; Martinsen), (C) ethical‐relational (Boykin & Schoenhofer), (D) cultural‐contextual (Leininger), (E) systemic‐organisational (Ray) and (F) operationalisable caring (Kolcaba; Swanson) (Table A4). This unified nomenclature reflects the capacity of these latter frameworks to objectify care into describable and evaluable structures without sacrificing its relational foundation. Consequently, the distribution into subfamilies is consistent with the dominant foundations and with the mechanism/*telos* synthesised for each group, reinforcing the thesis of an ordered plurality: the existence of a shared moral‐relational core that unfolds through non‐equivalent operative devices.

### 3.6 Metatheoretical Propositions Derived From the Comparative Analysis

Based on the systematic integration of definitions, *telos*, metaparadigmatic anchors and caring mechanisms, the following metatheoretical propositions were formulated, expressed as conditional relationships between assumptions, modes of care operation and expected effects.

When the person is conceptualised as an integral reality with a transpersonal or charitable horizon → caring operates primarily as intentional presence and moral relationship → effects linked to dignity, meaning, integrity and relief of suffering are expected.

When the person is understood as a situated and embodied subject in a concrete clinical situation → caring is deployed through clinical judgement and ethical perception → the appropriateness of care depends on practical competence and situated discernment.

When the environment is conceptualised as a constitutive cultural mediation → caring operates as a practice of cultural congruence → the validity of care is defined by its coherence with the values, meanings and practices of the group or context.

When the environment is conceptualised as an organisational and bureaucratic structure → caring operates as an ethical negotiation within technological, normative and economic mediations → the effects of care depend on institutional conditions that enable or erode practice.

When caring is formulated through explicit processes or taxonomic structures → care becomes translatable into teaching, planning and evaluation → expected effects include observable outcomes without necessarily eliminating the relational dimension.

When the *telos* of caring is defined as comfort or integral strengthening → care outcomes are legitimised through evaluable indicators → coherence depends on the measurement not reducing the patient's lived experience.

When caring is grounded in a strong relational ontology → the normativity of care precedes technique → there is a risk of abstraction if pedagogical, clinical, or organisational mediations are not explicated.

When caring is conceptualised as a co‐produced relational event → the minimum unit of analysis is the nursing situation → care is understood as an emergent moral event and not as a unilateral action.

When different configurations of caring share compatible mediations or differentiated levels of analysis → theoretical integration is plausible without conceptual reduction → an ordered disciplinary plurality is made possible.

When the explication of assumptions, mechanisms and moderators is omitted → caring tends to function as a rhetorical signifier → a gap is produced between ethical aspiration and effective practice.

## 4 Discussion

The aim of this study was to construct a comparative metatheory of the school of caring based on nine theories/models included in *Nursing Theorists and Their Work* (Alligood 2022): Watson; Boykin and Schoenhofer; Eriksson; Benner; Martinsen; Ray; Leininger; Swanson; and Kolcaba. Taken together, the results support the claim that this school is not a homogeneous block, but rather a theoretical field organised by shared invariants and systematic internal variations. The contribution of this metatheoretical analysis is twofold: (a) it delimits a common structural core—relationship, normativity, contextuality and transformative teleology—and (b) it explicates an internal architecture (typology and layered model) that links assumptions (ontological, epistemological and ethical), caring mechanisms and contextual moderators. This explication allows for comparison without reduction: it shows which part of the convergence is robust and which part depends on non‐interchangeable conceptual decisions between frameworks (Alligood 2022, 74, 88, 109, 131, 148–150, 294–295, 313, 340–342, 528, 530, 535, 556–557).

### 4.1 From ‘School’ to Metatheoretical Architecture: The Gains of Explicating Mechanisms

In everyday educational use, the ‘school of caring’ often functions as an affinity label: distinct theories are grouped together because they all assert, in some way, that caring is central. The metatheoretical problem with this label is that it homogenises from the top down: it recognises the centrality of caring but leaves its status (what kind of reality caring is), its logic (how it operates in the clinical encounter) and its translation (what conditions sustain it in practice, education and research) indeterminate.

The main contribution of the present work consists of transforming this affinity into an explanatory architecture: identifying invariants, describing differential dimensions and formulating caring mechanisms consistent with the assumptions of each theory. This step is crucial because the non‐interchangeability between frameworks does not depend on terminological nuances, but on how each theory grounds normativity, validates knowledge and delimits the relevant environment of care (Alligood 2022, 74, 88, 109, 131, 148–150, 313, 528–530, 556–557). Under this logic, the proposed formalisation is critical for disciplinary integrity for, as Proposition P10 warns, when the explication of assumptions and mechanisms is omitted, caring tends to function merely as a rhetorical signifier. By identifying invariants and differentiated operative mechanisms, this metatheory acts as the necessary antidote to close the gap between ethical aspiration and effective practice, preventing the concept from degrading into a ‘total’ signifier lacking heuristic capacity and professional utility.

In the nine theories analysed, caring delimits nursing beyond technical intervention: care is presented as a relationship oriented towards the integral person, with moral density and with outcomes that include dignity, relief of suffering, strengthening/comfort, or growth in care (Alligood 2022, 74, 109, 131, 148–150, 294–295, 528–530, 556–557). However, this high‐level agreement does not imply conceptual equivalence. The metatheoretical architecture shows that unity is sustained by a plurality of foundations: there is variation in (i) the source of normativity (e.g., *caritas*/transpersonality, relational responsibility, cultural congruence, right action in bureaucracy, comfort as strengthening), (ii) the status of legitimate knowledge (axiological‐experiential, clinical‐situated, cultural, organisational, evaluative), (iii) the level of analysis (micro‐relational encounter versus macro‐systemic mediation) and (iv) the degree of operationalisation (Alligood 2022, 74, 86–88, 109, 131, 313, 340–342, 528–530, 535, 556–557). Explicating these dimensions is what allows us to speak rigorously of a ‘school’: a family with a shared core but with differentiated mechanisms.

This reading aligns with contemporary analyses that describe nursing theory not as a unified system, but as a plural field articulated by shared conceptual cores and persistent structural differences (Meleis 2017).

### 4.2 Metatheoretical Synthesis of Caring: From Core Concept to Generative Principle

The most robust integrative finding is that, in this school, caring functions as a generative principle: it is not just a ‘content’ of nursing, but an ontological device that organises what counts as a person in vulnerability, what is recognised as health/well‐being, which environment is relevant and what is considered legitimate nursing practice (Alligood 2022, 74, 88, 109, 131, 148–150, 294–295, 313, 340–342, 528–530, 535, 556–557).

This principle manifests in a constant pattern: when the person is conceptualised as an integral reality irreducible to biomedical parameters, caring tends to be defined primarily as a moral relationship of recognition. Conversely, when the environment is explicated as a constitutive mediation (cultural or organisational), caring is interpreted as a situated practice whose coherence depends on competencies and the infrastructure of the health system. This metaparadigmatic reordering reinforces the thesis that caring is not a dependent variable, but the structuring axis that bestows meaning upon the other concepts of the discipline (Alligood 2022, 88, 109, 131, 340–342, 556–557).

This generative principle adopts two recurring metatheoretical configurations. In the first configuration, caring operates as an axiological‐ontological foundation: its legitimacy is derived from a strong normative horizon (e.g., transpersonality or *caritas*) and its effects are formulated in terms of dignity, meaning, integrity and relief of suffering (Alligood 2022, 74; pp. 148–150). In the second configuration, caring operates as a foundation translatable through mediations: it is expressed as embodied practical judgement (clinical wisdom), as cultural congruence, as negotiation/right action under institutional constraints, or as evaluable processes/outcomes (comfort, care processes) (Alligood 2022, 86–88, 109, 131, 313, 340–342, 528–530, 535, 556–557). The coexistence of both configurations explains a distinctive feature of the field: the school of caring is not defined in opposition to the empirical, but nor does it accept that measurement is the sole criterion of legitimacy; what is decisive is the internal coherence between assumptions, mechanism and the teleology of care.

From this perspective, integration does not imply total synthesis, but rather articulation by levels, consistent with pluralist approaches to theoretical development in nursing (Im and Chang 2012).

### 4.3 Metaparadigmatic Synthesis: Caring as an Operator Reordering Person, Health, Environment and Nursing/Care

A specific contribution of this metatheory is showing that, in the school of caring, the metaparadigm does not operate as four parallel concepts (person–health–environment–nursing), but as a system whose relationships are reordered by caring. Care does not ‘accompany’ person, health and environment; it determines the way of understanding them and of justifying nursing action. This thesis is supported by the fact that the metaparadigmatic fragments analysed in Alligood (2022) exhibit conceptual variations that are not accidental but consistent with the caring mechanism proposed by each theory. In particular, the concept of ‘environment’ emerges as the primary discriminant: its formulation makes visible the type of mediation that each framework considers constitutive (spiritual, situational, cultural, organisational, or manipulable for comfort) (Alligood 2022, 74, 88, 109, 131, 340–342, 528–530).

#### 4.3.1 Person: Integrality, Relationality and Non‐Reduction as a Condition of Care

In the nine theories, ‘person’ is separated from a reductive biomedical reading and formulated as an integral reality before which care acquires moral density. This integrality adopts variants consistent with each framework: spiritual‐unitary holism (Watson), body–soul–spirit anthropology and the centrality of dignity (Eriksson), embodiment as a way of perceiving and understanding (Martinsen), interpretive subject in a situation with social meaning (Benner), relational ontology of living and growing in care (Boykin & Schoenhofer), a being of care in a cultural key (Leininger), multiscalar recipients of needs (Kolcaba) and uniqueness in becoming with meaning (Swanson) (Alligood 2022, 74, 109, 131, 148–150, 294–295, 313, 340–342, 528–530, 556–557).

#### 4.3.2 Health: Non‐Neutral Teleologies and Consequences for Validation

The category of health/well‐being shifts towards formulations incorporating harmony, wholeness, meaningful experience, or strengthening. Watson links it to unity/harmony and congruence; Eriksson to integrity/totality; Benner and Martinsen differentiate the evaluable from lived well‐being, emphasising the situated nature of suffering and competent response; Leininger conceives it as culturally defined and valued well‐being; Ray inserts it into patterns of meaning mediated by organisation and values; Kolcaba associates it with optimal functioning and operationalises it through comfort as an integral experience of strengthening; and Swanson underlines meaningful well‐being within a life trajectory (Alligood 2022, 74, 88, 109, 131, 148–150, 340–342, 528–530, 556–557).

#### 4.3.3 Environment: Constitutive Mediations and the Shift From ‘Context’ to ‘Condition of Possibility’

The environment is the most discriminant concept due to its variation in scale and status: universal spiritual field (Watson), social situation with meaning (Benner), concrete lived space (Martinsen), culture as a determinant of care content (Leininger), bureaucratic‐technological organisation as a mediation of caring (Ray), or environment as a manipulable aspect to improve comfort (Kolcaba) (Alligood 2022, 74, 88, 109, 131, 340–342, 528–530). This variation forces an abandonment of the reading of caring as an exclusively individual virtue. Where the environment is defined as culture or organisation, care is a relational phenomenon whose coherence depends on mediations: cultural competence, context reading, ethical deliberation and institutional conditions that protect time, continuity and clinical presence (Alligood 2022, 86–88, 340–342, 556–557).

#### 4.3.4 Nursing/Care: From Technical Discipline to Situated Moral Praxis

In all theories, nursing is defined by a relational praxis with moral density; what varies is the mode of justifying it, training for it and making it visible. Across transpersonal, phenomenological and operationalisable frameworks, the diversity suggests that ‘nursing as care’ is a common proposition, but its operative content depends on which type of caring is prioritised and which mediations are considered legitimate (Alligood 2022, 74, 88, 109, 131–132, 148–150, 294–295, 340–342, 528–530, 556–557).

### 4.4 Plausible Compatibilities and Structural Tensions: Internal Translation Rules

A useful comparative metatheory does not only classify; it establishes criteria for deciding which integrations are plausible and which tensions are structural. The analysis suggests that the strongest compatibilities occur when two theories share a compatible mediation or when they are articulated at different levels without claiming equivalence. For example, cultural contextuality (Leininger) can be articulated with integral well‐being evaluation (Kolcaba) if comfort is interpreted as a situated experience and not a decontextualised universal outcome.

The model also makes visible tensions that must be managed through mediation, not conceptual ‘averaging’. The first tension is between ontological‐axiological density and operative translatability. The second tension is micro vs. macro: Ray forces the recognition that caring is negotiated in bureaucratic structures, meaning the moral quality of care depends as much on the encounter as on the system that enables presence.

### 4.5 Disciplinary Implications: Practice, Education and Research

In clinical practice, implementing caring requires explicating which configuration is prioritised. In education, the results support layered curricula that simultaneously train ethical competence, clinical judgement, cultural competence and organisational reading. In research, the metatheory provides a criterion for methodological alignment: the design must be congruent with the epistemology and teleology of the caring intended for study, avoiding category errors (e.g., measuring as an outcome what a theory defines as an irreducible moral relationship).

### 4.6 Strengths and Limits: The ‘Intra‐Source’ Reach as a Methodological Decision

A key strength is the coherence of the corpus: all theories proceed from the same text and edition (Alligood 2022). This criterion also delimits the scope: the resulting metatheory is, by design, an intra‐source synthesis. Its value lies in offering a transparent, replicable architecture for discussing conceptual coherence and disciplinary translation within the selected textual framework.

### 4.7 Conclusion of the Discussion: A Metatheory of Caring Configurations

Overall, the school of caring can be metatheoretically described as a field with clear invariants—relationship, normativity, contextuality and transformative teleology—and systematic variations in the source of normativity, the status of knowledge, the level of analysis and the degree of operationalisation. The proposed integrative model allows for precise discussion of plausible compatibilities and structural tensions, ensuring caring does not degrade into rhetoric or systemic compliance without encounter (Alligood 2022, 74, 86–88, 109–113, 131–132, 148–150, 294–295, 313, 340–342, 528–530, 535, 556–557).

## 5 Conclusions

This study developed a comparative metatheory of the school of caring based on nine theories/models presented in *Nursing Theorists and Their Work* (Alligood 2022). The final product of the synthesis is not a ‘merger’ of frameworks, but rather an architecture of ordered plurality that preserves the unity of the field without reducing its internal diversity. This architecture is sustained by: (a) an integrative metatheoretical model that organises convergences and divergences into levels (assumptions → metaparadigm → mechanisms → expected effects), identifying the variation in the scale of the environment as the primary discriminant axis of praxis; and (b) a set of metatheoretical propositions formulated as relational and conditional statements that link the ontology of care with clinical governance devices and disciplinary security.

Consequently, in terms of clinical practice, this model and its propositions function as an alignment map: it allows for the explication of which caring configuration is prioritised (assumptions, *telos* and mechanism) and the coherent derivation of intervention priorities and conditions of possibility (e.g., presence/continuity, cultural or organisational mediations, or evaluative criteria), reducing the risk of a merely rhetorical use of the term and reinforcing the disciplinary operationalisation of the ‘school of caring’.

The central contribution of this manuscript consists of metatheoretically operationalising the use of the ‘school of caring’. Rather than treating it as an affinity label, the analysis defines it as a theoretical family with recognisable invariants (relationship, normativity, contextuality and teleological orientation of care) and with stable structural variations that differentiate configurations of caring (source of normativity, status of knowledge, level of analysis and degree of operationalisation). This formalisation allows for the comparison of theories based on a criterion of non‐interchangeability: relevant differences are not merely terminological but depend on how each framework grounds the duty to care, validates knowledge and delimits the relevant environment.

Propositions P1–P10 are presented as non‐causal metatheoretical hypotheses: their function is to make explicit the logic connecting assumptions with caring mechanisms and expected consequences. In disciplinary terms, this provides an instrument to evaluate internal coherence (whether assumptions, metaparadigm, mechanism and *telos* ‘fit’) and to guide translation decisions (what can be integrated through compatibility of mediations or levels, and which tensions should be treated as structural and not resolvable by conceptual averages).

In summary, this manuscript offers a usable metatheory: it transforms ‘caring’ from a broad signifier into an object of comparative analysis with high strategic value for clinical governance. It provides explicit rules to (i) distinguish configurations of care, (ii) justify plausible integrations without reduction and (iii) derive coherent implications for teaching, practice and research. It is concluded that disciplinary maturity lies not in forced unification, but in the capacity to rigorously navigate theoretical plurality through transparent metatheoretical frameworks.

### 5.1 Main Contributions of the Manuscript

Delimits invariants and differential dimensions that convert the notion of the ‘school of caring’ into an operative metatheoretical object for internal comparison.

Proposes a typology of subfamilies (configurations) that explains plausible compatibilities and structural tensions without reducing theories to a common denominator.

Integrates a layered model linking the metaparadigm, assumptions (ontology/epistemology/ethics), caring mechanisms and contextual moderators, providing conceptual traceability.

Formulates metatheoretical propositions (condition → mechanism → effect) usable for guiding congruent decisions in practice, teaching and research.

### 5.2 Implications

Clinical Practice. Implementing caring requires explicating the prioritised configuration (e.g., transpersonal/caritas; situated judgement and embodiment; relational recognition; cultural congruence; organisational mediation; processes or comfort) and protecting the corresponding moderators (presence/continuity, contextual competence, ethical deliberation in the organisation, or evaluative infrastructure), avoiding the rhetorical use of the term (Alligood 2022, 88, 109, 131, 313, 340–342, 528, 530, 556–557).

Education. The teaching of caring benefits from a layered curriculum, aligning ethical and relational training with training in situated clinical judgement, cultural competence, organisational reading of care and literacy in the evaluation of processes/outcomes consistent with integrality (Alligood 2022, 88, 109, 131, 313, 528, 556–557).

Research. The model suggests differentiated programmes: approaches sensitive to experience and situation for phenomenological‐embodied frameworks; strategies centred on meanings and cultural patterns for Leininger; incorporation of institutional moderators for Ray; and evaluative designs when caring is formulated as processes or comfort (Alligood 2022, 88, 109, 131, 340–342, 528, 530, 556–557).

### 5.3 Limitations and Future Lines of Enquiry

The scope of the study is deliberately intra‐source, an epistemological decision to ensure comparative consistency. The resulting metatheory describes caring as codified and canonised in the selected base text. Future lines of enquiry consist of: (a) extending the analysis towards an intertextual comparison with the original works of each theory to evaluate correspondences, divergences and conceptual evolution; and (b) empirically contrasting how organisational and cultural moderators affect the practical translation of caring in real contexts (Alligood 2022, 88, 313, 340–342, 528, 530, 556–557).

#### 5.3.1 Prioritised Future Lines

Expansion of Intertextual Triangulation: Perform the analysis on the complete corpus of the original works of each theory to verify correspondences and divergences regarding the Alligood chapter.

Research Programmes by Configuration: Design studies consistent with the epistemology of each subfamily (e.g., evaluative for comfort/processes; experience‐centred studies for situated judgement; systems analysis for bureaucracy).

Moderators as Critical Variables: Operationalise and measure institutional conditions (time, continuity, bureaucratic load) and cultural conditions (congruence) that enable or erode caring.

## Author Contributions

**Aarón Muñoz Devesa:** conceptualization; data curation; formal analysis; methodology; writing – original draft; writing – review and editing. **Juan Ignacio Rico Becerra:** conceptualization; supervision; methodology; critical revision of content; writing – review and editing.

## Funding

The authors have nothing to report.

## Disclosure

IRB statement: This study is a documentary analysis of published sources and does not involve human participants or identifiable clinical data; therefore, it did not require approval from an ethics committee/IRB.

## Ethics Statement

The authors have nothing to report.

## Consent

The authors have nothing to report.

## Conflicts of Interest

The authors declare no conflicts of interest.

## Data Availability

The data that support the findings of this study are available from the corresponding author upon reasonable request.

## Appendix A

**TABLE A1:** Taxonomy of caring configurations: semantic families and textual evidence in the school of caring.

Theorist/theory	Semantic family (metatheory)	Concept focus	Identifying fragment [page]
Jean Watson	Transpersonal/caritas	Caring/care	«Human caring relationship (the union with another person) with a special regard for the whole person…» [70]
Katie Eriksson	Transpersonal/caritas	Caring/care	«Charitable care means that we use caritas when we care for the human being in health and suffering.» [143]
Patricia Benner	Phenomenological‐embodied/clinical wisdom	Caring/care	«Caring relationship, a “condition of connection and concern”». [109]
Kari Martinsen	Phenomenological‐embodied/clinical wisdom	Caring/care	«Trinity: relational, practical and moral simultaneously. Care is directed towards the situation in which the other person finds themselves.» [129]
Anne Boykin & Savina O. Schoenhofer	Ethical‐relational	Caring/care	«Altruistic and active expression of love, and it is the intentional and embodied recognition of value and connection.» [301]
Madeleine M. Leininger	Cultural‐contextual	Caring/care	«Essence and the central dominant, distinct and unifying focus of nursing» (emphasis on cultural congruence). [341]
Marilyn Anne Ray	Systemic‐organisational	Caring/care	«Complex transcultural and relational process… relationship between charity and right action» (institutional mediation). [87]
Katharine Kolcaba	Operationalisable	Comfort	«Immediate state experienced by recipients… immediate and integral experience of being strengthened.» [528]
Kristen M. Swanson	Operationalisable	Caring/care	«Nurturing way of relating… towards which one feels a personal sense of commitment and responsibility.» [556]

**TABLE A2:** Teleological orientations of caring: typology of purposes and internal goods by theoretical family.

Theorist/theory	Subfamily (typology)	Concept focus	Identifying fragment (telos) [page]
Jean Watson	Transpersonal/caritas	Caring/care	«Promote and enhance human dignity, wholeness and healing, allowing the person to create their own sense of existence.» [71]
Katie Eriksson	Transpersonal/caritas	Caring/care	«Caring communion… occurs when the one who cares in the spirit of caritas alleviates the patient's suffering.» [143]
Patricia Benner	Phenomenological‐embodied	Caring/care	«Caring is primary because it sets up the possibility of giving and receiving help.» [109]
Kari Martinsen	Phenomenological‐embodied	Caring/care	«Caring is the positive development of the person through the Good. […] A fundamental prerequisite for our life.» [129]
Anne Boykin & Savina O. Schoenhofer	Ethical‐relational	Caring/care	«Nurturing persons living caring and growing in caring.» [291]
Madeleine M. Leininger	Cultural‐contextual	Caring/care	«Provide culturally congruent care… so that they maintain or regain their well‐being or health, or face death.» [339]
Marilyn Anne Ray	Systemic‐organisational	Caring/care	«Spiritual‐ethical caring… focuses on facilitating choices for the good of others» [within institutional mediations]. [87]
Katharine Kolcaba	Operationalisable	Comfort	«Strengthens patients to engage in health‐seeking behaviours of their choice.» [529]
Kristen M. Swanson	Operationalisable	Caring/care	«To promote their dignity, respect and empowerment.» [559]

**TABLE A3:** Mapping of metaparadigmatic anchors in the school of caring: comparative analysis of identifying fragments.

Theorist/theory	Subfamily	Metaparadigm element	Identifying fragment (evidence of scale and status) [page]
Jean Watson	A. Transpersonal	Environment	«We belong to an infinite universal spiritual world… primordial link between humanity and life itself.» [74]
Jean Watson	A. Transpersonal	Person	«Unity of mind/body/spirit/nature… the soul possesses a body that is not limited by time and space.» [74]
Katie Eriksson	A. Transpersonal	Environment	«Caring culture… describes the total caring reality and is based on cultural elements such as traditions.» [147]
Katie Eriksson	A. Transpersonal	Health	«Implies being whole in body, soul and spirit. […] wholeness and holiness… a becoming, a movement towards totality.» [149–150]
Patricia Benner	B. Phenomenological	Environment	«Use the term situation… because it conveys a social environment with a social definition and meaning.» [109]
Patricia Benner	B. Phenomenological	Nursing	«Caring relationship, a “condition of connection and concern”… sets up the possibility of giving and receiving help.» [109]
Kari Martinsen	B. Phenomenological	Environment	«Concrete situation and in a concrete space. […] The sickroom is the place where time stands still.» [131–132]
Kari Martinsen	B. Phenomenological	Person	«The body is a unit of soul and flesh. […] The person is corporeal, and as bodies, we perceive and also understand.» [131]
Boykin & Schoenhofer	C. Ethical‐Relational	Nursing	«Nurturing persons living caring and growing in caring. […] Altruistic and active expression of love.» [294, 301]
Boykin & Schoenhofer	C. Ethical‐Relational	Environment	«The nursing situation… is the shared and lived experience in which the caring between the nurse and the patient occurs.» [292]
Madeleine Leininger	D. Cultural	Environment	«The totality of a particular event, situation, or experience that gives meaning to expressions… the environmental context.» [340]
Madeleine Leininger	D. Cultural	Person	«Human beings as inseparable from their cultural backgrounds and social structure factors.» [343]
Marilyn Anne Ray	E. Sistemic	Environment	«Complex spiritual, ethical, ecological and cultural phenomenon… encompasses community and bureaucratic organisations.» [88]
Marilyn Anne Ray	E. Sistemic	Nursing	«Holistic care… that seeks the good… in community, organisational and bureaucratic cultures.» [88]
Katharine Kolcaba	F. Operational	Environment	«Any aspect… that can be manipulated by nurses… to enhance comfort.» [530]
Katharine Kolcaba	F. Operational	Health	«Optimal functioning of a patient… as defined by the patient or group.» [530]
Kristen Swanson	F. Operational	Environment	«Defines the environment as situational. […] any context that influences or is influenced by the client.» [557]
Kristen Swanson	F. Operational	Health	«To experience health and well‐being is to “live the subjective and meaningful experience of wholeness.”» [557]

**TABLE A4:** Taxonomy of the school of caring: Subfamily configuration by foundation, mechanism and telos.

Family	Label	Theorists	Dominant foundation	Mechanism and telos (synthesis)
A	Transpersonal Caring/Caritas	Watson; Eriksson	Ontological‐axiological foundation (transpersonality/caritas).	Relational encounter with spiritual horizon; health as harmony/integrity; telos: dignity and relief of suffering.
B	Phenomenological‐Embodied Caring & Clinical Wisdom	Benner; Martinsen	Clinical prudence and situated understanding (embodiment/experience).	Competent practical judgement; relational‐practical‐moral integration; environment as lived situation/space.
C	Ethical‐Relational Caring	Boykin & Schoenhofer	Relational ontology of care (living/growing in care).	Embodied recognition of value and connection; telos: human growth in care.
D	Cultural‐Contextual Caring	Leininger	Cultural congruence as a condition of validity.	Cultural patterns and meanings determine care content; telos: culturally congruent well‐being.
E	Systemic‐Organisational Caring	Ray	Right action in structures (bureaucracy, economy, law, technology).	Institutional mediation of caring values; tension between system and spiritual‐ethical care.
F	Operationalisable Caring	Kolcaba; Swanson	Translation into processes/outcomes without losing integrality.	Comfort/strengthening (Kolcaba) and relationship with commitment/responsibility (Swanson); facilitates implementation and evaluation.

**TABLE A5:** Caring mechanisms by theory/model (identifying fragments, evidence and analytical note).

Theory/model (Alligood 2022)	Mechanism family	Caring mechanism (analytical label)	Identifying fragment (ID)	Page	Literal core quote (minimum evidence)	Analytical note (interpretive synthesis)
Jean Watson	F1	Caritas processes (procedural structure of caring)	WAT‐CP	74	«…intentional encounter sustained by moral and relational consciousness»	Caritas processes operate as an intentional guide for the care encounter; they organise action in a moral‐relational key.
Marilyn Anne Ray	F2	Dimensions of bureaucratic caring as systemic mediators	RAY‐BC	86	«…dimensions of bureaucratic caring [economic, political, legal, technological] that function as mediators of care»	Caring is negotiated within institutional mediations (technological, legal, economic, etc.); the system conditions the possibility of care.
Patricia Benner	F3	Situated clinical judgement as a mechanism of practical discernment	BEN‐JC	113	«…mechanism that allows interpreting the situation, discerning priorities and responding appropriately»	Expert caring is deployed by interpreting the situation and prioritising actions; it integrates practical knowledge and a morally adequate response.
Kari Martinsen	F3	Ethical perception/attention as openness to the other	MAR‐PA	132	«…ethical perception—sensitive to the situation and the other—opens the possibility of care»	Moral‐sensitive perception guides care from the concrete situation; attention is the basal gesture that enables the encounter.
Katie Eriksson	F4	Confirmation of suffering as a constitutive act of charitable care	ERI‐CS	150	«…confirmation of the patient's suffering as a constitutive act of charitable care»	Caring operates by recognising and confirming suffering; it grounds dignity and relief through caritas.
Boykin & Schoenhofer	F5	‘Nursing situation’ as a co‐produced minimum unit	B&S‐SN	292	«…expression of values, intentions and actions of two or more persons [in the nursing situation]»	Caring is understood as an emergent relational event; it is analysed as a shared situation of intentions/actions.
Madeleine Leininger	F1	Three modes of action (preservation–accommodation–restructuring) for congruent care	LEI‐3M	342	«…three modes of action—preservation, accommodation and restructuring—that organise care»	The mechanism translates caring into culturally congruent decisions; culture is a constitutive mediation.
Katharine Kolcaba	F1	Taxonomic structure of comfort: relief–ease–transcendence	KOL‐CONF	535	«…states of comfort as a result of care: relief, ease and transcendence»	Caring is operationalised into comfort states/dimensions as an integral outcome, susceptible to planning and evaluation.
Kristen Swanson	F1	Caring processes (e.g., ‘being with’, ‘maintaining belief’, etc.)	SWA‐5P	556–557	«…relational processes [“being with”, “maintaining belief”] that guide action and emotional presence»	Caring is deployed as a set of relational processes that guide intervention and support; it promotes empowerment/dignity.

*Note:* F1 = explicit processes; F2 = institutional mediations; F3 = situated epistemic‐moral operations; F4 = suffering/confirmation; F5 = nursing situation.
